# General Medicine and Therapeutics

**Published:** 1896-04

**Authors:** 


### GENERAL MEDICINE AND THERAPEUTICS.

EDITED BY DR. LUTHER B. GRANDY.

### STYLE IN MEDICAL WRITING.

In a late address to medical students (*Journal of American Medical Association*) Dr. Edmund Andrews, of Chicago, gave expression to some excellent ideas on this subject:

It is amusing and yet vexatious to see a worthy medical gentleman whose ordinary conversation is in a simple and good style, suddenly swell up when he writes a medical article. He changes his whole dialect and fills his pages with a jangle of harsh technical terms, not one-third of which is necessary to express his meaning. He tries to be solemn and imposing. For instance, a physician recently devised a new instrument, and wrote it up for a medical journal under this title, "A new Apparatus for the Armamentarium of the Clinician."

Another writer wanted to say that cancer is an unnatural growth of epithelium. He took a big breath and spouted the following: "Carcinoma arises from any subepithelial poliferation by which epithelial cells are isolated and made to grow abnormally." Now, then, you know all about cancer.

The following is an effort to say that certain microbes produce the poison of erysipelas: "The streptococcus erysipelatosus proliferating in the interspaces of the connective tissues is the etiologic factor in the secretion of the erysipelatous toxins."

A large cancer of the liver was found at a post-mortem examination and reported about as follows: "A colossal carcinomatous degeneration of the hepatic mechanism."

Your man of solemn speech is peculiar. He does not keep a set of instruments—not he—he has an armamentarium. His catheters never have a hole or an eye in them, but always a fenestrum. In gunshot injuries, a bullet never makes a hole in his patient, but only a perforation. He does not disinfect his armamentarium by boiling, but by submerging it in water elevated to the temperature of ebullition. He never distinguishes one disease from another, but always differentiates or diagnosticates it. His patient's mouth is an oral cavity. His jaw is a maxilla. His brain is a cerebrum, his hip-joint is a coxofemoral articulation. If his eyelids are adherent, it is a case of ankylo-symblepharon. If he discovers wrinkles on the skin, they are corrugations or else rugosities. He never sees any bleeding, but only hemorrhage, or sanguineous effusion. He does not examine a limb by touch or by handling—he palpates or manipulates it. If he finds it hopelessly diseased he does not cut it off—that is undignified. He gets out his armamentarium and amputates it.

A good British surgeon has good culture and good common sense combined. He takes his trocar and taps a dropsy, for instance. Not so the American. He blushes to hear such plain speech from his British brother, but he takes his armamentarium and goes out straightway and performs paracentesis abdominis. Still the pompousness is not all on one side of the ocean, and it is refreshing to see an occasional touch of it beyond the seas, just to show that we are not the only sinners in this respect.

A celebrated British surgeon explains his opinions as follows: "Septic peritonitis, save where definable from evidence wholly extrinsic to the condition of the peritoneum, is an etiologic entity which exists only in the mind of the pathologic metaphysician." Nothing worse than this was ever written on our side of the water.

Two of the stilted phrases which have recently gotten into vogue are etiologic factor and etiologic entity. They are both snobbish substitutes for the plain English word, cause.

Such a language is the pure English of to-day. Derived mainly from the Anglo-Saxon, its words are chiefly monosyllables, but they are all gems, and all polished and fitted to each other by ages of use. Whoever is master of it, is an artist in the highest sense. Whoever thrusts into it a rough, harsh, polysyllabic word, crudely quarried out of a Greek or Latin dictionary, is like a fool who drives a pickaxe through a magnificent mosaic picture, and then plugs the hole with a brickbat.

The author who dumps upon us a book full of long and ragged new words, merely to display his knowledge of ancient languages, shows his ignorance of English, and brands himself as a bungler and a literary blockhead.

As I said before, use newly coined words sparingly, but when they become necessary, select them as carefully as you would choose jewels for your bride. Fit them, polish them, and make them suitable for flashing forth the best thoughts of human souls.

It is curious that the half-educated man who knows little or nothing of the classics, always dotes most on the big phrases, and is ever foremost in piling up the abominable rubbish-heap of long, scraggy words which deform medical literature, and render it a melancholly spectacle to gods and men.

The finer culture of the future will demand a reorganization of our word system, and will cast to the moles and the bats very much worthless material which is now the pride of the ignoramuses who invented it. Prepare yourselves therefore for the change, and do your best to make the language of future science more bright, pure and simple, and more fit for the use of all who wish to couple clearness of thought with beauty of expression.

---

### FEIGNED INSANITY.

The *Journal of Nervous and Mental Diseases* for March contains an abstract of a good paper by Dr. Jno. B. Chapin, Philadelphia, on this subject, and it is appropriate that we reproduce part of it at this time. The paper was an outline sketch of eight cases of feigned insanity that had come under the author's observation :

"All of these criminals were habitual law-breakers, or had been the associates of criminal and depraved persons. Two were under sentence of death ; one had committed a homicide; one had assaulted with an attempt to kill; one was under indictment for bigamy; and one was a thief and professional feigner. All had committed crimes and feigned insanity after conviction and sentence, or after indictment. All had had opportunities of seeing insane persons—were possessed of some degree of low cunning, and probably had the faculty of imitation. Three showed some of the common manifestations of advanced dementia, and one epilepsy. They affected these forms as probably requiring less physical exertion and mental strain. Two appeared to be maniacal for a brief period; and two showed no characteristic symptoms of any form of insanity—their acts being in the nature of small tricks. In two of the cases, the earliest manifestations that attracted attention were howling and shouting in the night, and throwing torn clothing and bedding out of the cell doors. In every case the simulation began suddenly, and presented forms of mental disorder that require ordinarily for their development the lapse of months and years. There was in every case a sufficient motive to resort to any effort or scheme to escape punishment.

"It is the usual experience that the symptoms presented to the physician are demonstrative, pronounced, and such as to attract attention at once. This was true in all these cases. This may arise from the belief that prevails among the common people and the inexperienced, that insanity appears suddenly, and is accompanied by some sudden and marked change in the individual. As a rule, I have found it difficult or impossible to obtain any satisfactory history of these cases prior to the commencement of the supposed attack. It is of rare occurrence that the case has even come under the intelligent observation of a jail physician prior to the outbreak, or indeed, of any person but a guard or some perfunctory attendant; so that the physician is thrown mainly on his own resources for information, and must frequently contend with unwilling witnesses. It has been my experience usually, to be unable to obtain from any physician or any person related to the case, any notes of observation, or any facts having any bearing on the case.

"If a physician in the course of his official duty is called in the interest of public duty and justice to examine and give testimony in relation to a person suspected of feigning, how shall he proceed to perform the service? In this direction he will obtain few or no suggestions from medical literature, as it is limited in amount and extent, and, as has been stated, he will derive little aid from persons in any way related to the case, and the history of the case will probably be limited and perhaps unreliable.

"In the first place, while he knows from observation and experience that there is a uniformity of forms and types of mental disorder, it will be found that every one of these shammers present some peculiarity that is a separate and special study—just as men differ from each other in their normal conditions, and as cunning and practised villains will devise new schemes to aid them in their simulation.

"While the physician must approach the examination of these cases without bias, and with such judicial calmness as he can command, he must take into account the fact that a dominating motive exists in the mind of the prisoner or suspect to deceive, for it is a part of the case.

"As to any rule or tests of precision to be followed in these cases, I know of none but those determined by experience and observation. Every physician concedes that the cases he meets in the ordinary line of duty proceed according to some recognized course of development. For instance, he does not look for a characteristic typhoid eruption nor developed small-pox on the first day of the sickness. If he does find these indications on his first visit he properly concludes there have been developmental or prodromal stages which have passed, and he can even name the day of the duration of the disease. There is an order of development of all diseases which constitute a rule; if there are exceptions, they must be accounted for on the hypothesis that they are instances of the limitations of our present knowledge.

"It is true also of mental cases that in accordance with a rule as infallibly fixed as in the diseases the general practitioner deals with, there is a general and uniform order of development in every case. Observation and uniformity of experience makes the rule, though there may be exceptions here, which is but another term to indicate that there are limitations of our actual knowledge. If, for example, a man on trial is able to sit with his counsel, suggest questions and a line of defense, making an allegation of insanity at the time of the homicide or during his trial—preferring to show that he acted in self-defense—and, when convicted, shall suddenly become reticent, saying he was engaged upon great inventions and did not wish to be disturbed; manifesting generally delusions of suspicion and fixed ideas, such as belong to the terminal stage of chronic insanity—we are unable to recognize a prodromal or primary stage. This is incompatible with general experience. We cannot reconcile the inconsistencies. The case in a usual order of procedure is thus exactly reversed. Yet referring to this case and to others, we see a man whom to look upon is observed to be doing what insane persons actually do. He is engaged, however, like the actor in the play—doing a part—in itself, perhaps, admirably done. It is then for the physician to close his eyes, and on reviewing the whole, to determine whether it is in accord with his actual clinical experiences. Acute mania, or apparently complete or stuporous dementia, do not develop in a day to a degree that weeks and months of disease are actually known to be necessary to produce. The terminal stage usually does not take precedence of the primary.

"It may be said, then, in conclusion, that no ascertained test of determining real from feigned insanity absolutely exists, except the rule of experience and the results of observation. The physician must enter upon the examination of these cases free from bias, with a judicial temper, about as he would proceed to form an opinion of any obscure case that might present in his every-day experience. For myself, I have usually appeared to those persons casually, and engaged them in conversation, if possible, or observed them when they refused to talk. I have never concealed the object of my visit when asked, nor my profession, but have announced it before the visit finally terminated. The endeavor should be made, I think, to ascertain the existence of consistencies, or inconsistencies; to ascertain the presence of bodily complications, and constitutional sympathetic disturbances—disturbances such as might be looked for to make out a symmetrical whole. After all efforts to reach a satisfactory conclusion have been exhausted, even after sufficient time which is essential has been allowed to elapse, it is still possible that human judgment may err, but it is not probable."

---

### PRACTICAL SUGGESTIONS TO MEDICAL EXPERTS.

Hon. Tracy C. Becker, Professor of Medical Jurisprudence in the University of Buffalo, has suggested the following practical rules and admonitions for medical witnesses:

First. A physician should refuse to testify as an expert unless he is conscious that he is really qualified as an expert.

Second. After accepting the responsibility, his first duty should be to make a diligent examination and preparation for his testimony, unless it is upon a subject with which he is familiar and which he is satisfied he has already exhausted, by reading the best authorities he can find, and by careful reflection upon particular questions as to which his opinion will be asked.

Third. Where he is to make an examination of facts, such as the post-mortem examination of a body, a chemical analysis, or an examination of an insane person, he should insist upon having plenty of time and full opportunity for doing his work thoroughly. He should take particular pains to make his examination open and fair, and, if possible, should invite opposing experts to co-operate with him in it.

Fourth. He should be honest with his client before the trial in advising him and giving him opinions, and upon the trial should preserve an absolutely impartial attitude, concealing nothing, perverting nothing, exaggerating nothing.

Fifth. On the preliminary examination as to his qualifications as a witness, he should be frank and open in answering questions. He should state fully the extent and the limits of his personal experience and of his reading upon the subject, without shrinking from responsibility, yet without self-glorification.

Sixth. He should be simple, plain, and clear in his statement of scientific facts and principles, avoiding the use of technical language, and trying to put his ideas in such form that they will be grasped and comprehended by men of ordinary education and intelligence.

Seventh. He should avoid stating any conclusions or principles of which he is not certain, but having an assurance that he is right, he should be firm and positive. He should admit the limitations of his knowledge and ability. Where a question is asked which he cannot answer he should not hesitate to say so; but he should refuse to be led outside the subject of inquiry, and should confine his testimony to those scientific questions which are really involved in the case or in his examination of the case.

Eighth. And, finally, he should always bear in mind that at the close of his testimony an opportunity is usually given to him to explain anything which he may be conscious of having said which requires explanation; and partial statements which need a qualification to make them a truth. This is the physician's opportunity to set himself right with the court and with the jury. If the course of the examination has been unsatisfactory to him, he can then, by a brief and plain statement of the general points which he has intended to convey by his testimony, sweep away all the confusion and uncertainty arising from the long examination and cross-examination, and can often succeed in producing for the first time the impression which he desires to produce, and can present the scientific aspects of the case briefly and correctly.

---

### GONORRHEA.

Dr. Martin gives the following prescriptions as the best and most effective of some several thousands examined and used by him in the treatment of gonorrhea. Early stage:

| | | |
|---|---|---|
| ℞ | Acidi carbolici | m. xx. |
| | Opii aqueosi extr | gr. xx. |
| | Acidi boraci | dr. ss. |
| | Liq. plumbi acetatis q. s. ad | fl. oz. vi. |
| ℞ | Hydrarg. chlor. corros | gr. ⅛. |
| | Sodii chlor | dr. j. |
| | Aquæ | fl. oz. vi. |
| ℞ | Potass. permanganatis | gr. ½. |
| | Sodii chlor | dr. j. |
| | Aquæ | fl. oz. vi. |
| ℞ | Argent nitrat | gr. j. |
| | Aquæ | oz. j. |

Sig. Use gtt x in a tablespoonful of water.

| | | |
|---|---|---|
| ℞ | Acidi carbolici | gr. xxx. |
| | Sodii chlor | dr. j. |
| | Aquæ, q. s. ad | fl. oz. vi. |
| ℞ | Potass. permanganatis | dr ss. |
| | Aquæ | fl. oz. iij. |

Sig. Add two teaspoonfuls to a quart of water, to which is added two teaspoonfuls of salt. Use in a fountain syringe and irrigate bladder.

In all of the preceding prescriptions, except the last, use one syringe full to wash out the urethra and allow it to flow out at once. Then use another syringe full and keep this in contact with the membrane for three minutes before allowing to escape. For the last stage:

| | | |
|---|---|---|
| ℞ | Zinci acetatis | gr. xxx. |
| | Acidi tan | gr. xxx. |
| | Aquæ rosæ | fl. oz. vi. |
| ℞ | Extr. hydrastis fluidi, | |
| | Bismuthi subcarbonatis | aa. dr. ij. |
| | Glycerini | fl. dr. ij. |
| | Aquæ, q. s. ad | fl. oz. vi. |
| ℞ | Zinci sulph. | |
| | Aluminis (pulv.) | aa. gr. v. |
| | Acidi carbolici | gr. iv. |
| | Aquæ, q. s. ad | fl. oz. vi. |
| ℞ | Zinci sulphatis | gr. xv. |
| | Plumbi subacetatis | gr. xx. |
| | Tinct. opii | fl. dr. ij. |
| | Tinct. catechu | fl dr. j. |
| | Aquæ, q. s. ad | fl. oz. vi. |

Internally the doctor administers:

| | | |
|---|---|---|
| ℞ | Bals. copaibæ, | |
| | Oleoresinæ cubebæ, | |
| | Pepsin, | |
| | Salol | aa. gr. v. |

Misce et ft. capsulam j.
Sig. Two capsules after each meal.

| | | |
|---|---|---|
| ℞ | Ol sandal | m. v. |
| | Bals. copaibæ | m. v. |
| | Ol. cinnamoni | m. j. |

Misce et ft. capsulam j.
Sig. Two capsules after each meal.

In all applicable cases the doctor prefers the method of irrigation by the fountain syringe, the drawback being the difficulty of keeping the permanganate solution from splashing from the urethra and staining the clothes of the physician who administers the douche.—*Medical World.*

---

### DIPHTHERIA ANTITOXIN FROM THE CLINICIAN'S STANDPOINT.

At a recent meeting of medical practitioners held in New York city, Dr. A. Caillé opened a discussion regarding the present status of the diphtheria antitoxin viewed from a clinical standpoint. He presented a brief up-to-date *résumé* of the subject, and offered some suggestions as to the clinical indications for the exhibition of Behring's remedy.

Caillé stated that Behring's antitoxin has now been on trial for more than two years over the entire civilized world, and, after every possible allowance for exaggeration and error has been made, the fact remains that the discovery of Behring is one of the greatest of modern times.

The precise action of antitoxin, Caillé said, is not known; but the same may be said of iodide of potassium, antipyrin, thyroid extract, mercury, and many other remedial agents whose beneficial and curative effects are universally recognized. From a study of the voluminous literature and also from what personal experience has taught, it is admitted that occasionally an exanthem or joint swelling follows the subcutaneous administration of antitoxin. Such manifestations are not sufficiently grave to contraindicate the employment of the remedy, for they are not attended with danger to life.

More than 100,000 injections of serum antitoxin have been made, and no reliable proof has been established that in a single instance death could be directly attributed to the treatment.

The author believes that a study of all cases goes to show:

1. That serum antitoxin has a specific curative and immunizing action.

2. That the frequency of complicating nephritis in cases of diphtheria treated by antitoxin is diminished as compared with diphtheria cases without specific treatment.

3. That the frequency of albuminuria and post-diphtheritic paralysis appears to be about the same as before the introduction of the serum therapy.

4. The prevention and cure of laryngeal diphtheria without intubation or tracheotomy, and the shortening of the period for wearing the tube in instances demanding such interference, are among the most striking and convincing results of the specific treatment.

5. Supplementary measures (stimulation and *mild* local treatment) must not be neglected.—*Am. Med.-Surg. Bulletin.*

